# Color-resolved Cherenkov imaging allows for differential signal detection in blood and melanin content

**DOI:** 10.1117/1.JBO.28.3.036005

**Published:** 2023-03-13

**Authors:** Vihan A. Wickramasinghe, Savannah M. Decker, Samuel S. Streeter, Austin M. Sloop, Arthur F. Petusseau, Daniel A. Alexander, Petr Bruza, David J. Gladstone, Rongxiao Zhang, Brian W. Pogue

**Affiliations:** a Dartmouth College, Thayer School of Engineering, Hanover, New Hampshire, United States; b Dartmouth College, Geisel School of Medicine, Department of Medicine, Hanover, New Hampshire, United States; c University of Wisconsin–Madison, Department of Medical Physics, Madison, Wisconsin, United States

**Keywords:** Cherenkov, Cerenkov, radiotherapy, luminescence, radiation

## Abstract

**Significance:**

High-energy x-ray delivery from a linear accelerator results in the production of spectrally continuous broadband Cherenkov light inside tissue. In the absence of attenuation, there is a linear relationship between Cherenkov emission and deposited dose; however, scattering and absorption result in the distortion of this linear relationship. As Cherenkov emission exits the absorption by tissue dominates the observed Cherenkov emission spectrum. Spectroscopic interpretation of this effects may help to better relate Cherenkov emission to ionizing radiation dose delivered during radiotherapy.

**Aim:**

In this study, we examined how color Cherenkov imaging intensity variations are caused by absorption from both melanin and hemoglobin level variations, so that future Cherenkov emission imaging might be corrected for linearity to delivered dose.

**Approach:**

A custom, time-gated, three-channel intensified camera was used to image the red, green, and blue wavelengths of Cherenkov emission from tissue phantoms with synthetic melanin layers and varying blood concentrations. Our hypothesis was that spectroscopic separation of Cherenkov emission would allow for the identification of attenuated signals that varied in response to changes in blood content versus melanin content, because of their different characteristic absorption spectra.

**Results:**

Cherenkov emission scaled with dose linearly in all channels. Absorption in the blue and green channels increased with increasing oxy-hemoglobin in the blood to a greater extent than in the red channel. Melanin was found to absorb with only slight differences between all channels. These spectral differences can be used to derive dose from measured Cherenkov emission.

**Conclusions:**

Color Cherenkov emission imaging may be used to improve the optical measurement and determination of dose delivered in tissues. Calibration for these factors to minimize the influence of the tissue types and skin tones may be possible using color camera system information based upon the linearity of the observed signals.

## 1 Introduction

Radiotherapy is used in ∼50% of cancer patients’ therapy.^1^^,^^2^ Few measurement devices such as thermoluminescent dosimetry or optically stimulated luminescent dosimetry exist to verify dose to tissues and are usually limited to point dose determination. At present, no available technique can quickly and readily provide information regarding the dose distribution as well as the beam position and strength in real time;^3^ however, prior work has pioneered the implementation of Cherenkov emission imaging as a quality assurance tool in photon and electron beam radiation therapy.^4^

Cherenkov radiation is the visible-light emitted when charged particles, such as electrons, pass through a dielectric medium traveling at a speed greater than the phase velocity of light.^5^ This leads to an electromagnetic interaction for emission, commonly perceived as a blue light in clear media such as water. The emission is in fact broadband and covers the entire UV/visible/IR spectrum, but is of maximal intensity in the blue.^6^ The Cherenkov signal is directly proportional to the deposited dose because it results from the soft collisions of the electrons with the medium.^6^ The practical application of this observation is that the signal might be used as a means of quantitatively mapping radiation dose in humans. The exploration of this physical phenomenon within a therapeutic setting is a major aspect of this study.

Cherenkov imaging is a useful tool to visualize the beam shape on tissue, but when used as a tool for dosimetry, it requires additional study at a fundamental level to potentially overcome quantitative limitations. Generation of Cherenkov light is directly related to dose, radiation energy spectrum, and the local refractive index. Transportation of this light is affected by attenuation from the intrinsic tissue optical properties. Monte Carlo simulations have predicted that tissue absorption and scattering events may contribute up to 45% variation in the detected light, and skin color change could alter the signal level by 90%.^1^^,^^7^ Experimental results have shown that in tissue phantoms, varying the content of blood and Intralipid can result in a difference of up to 20% in surface Cherenkov emissions.^8^ The study here was focused on determining the differences between individual tissue types in the context of Cherenkov emission intensity, to further the potential for dosimetric information.^9^

In this study, color Cherenkov imaging was accomplished with a custom three-channel camera, that had time-gated image intensifiers on each color channel [red, green, and blue (RGB)]. Time-gated imaging allowed for the detection of low-intensity Cherenkov emission signal above background ambient light levels by acquiring image frames only when linear accelerator beam pulses were present.^10^^,^^11^ Cumulative images of color Cherenkov emission were captured from tissue phantom samples irradiated with megavoltage (MV) x-rays from a clinical linear accelerator (LINAC). This imaging setup was used to explore the hypothesis that multi-spectral color Cherenkov imaging could allow for differential signal detection in tissues with variations in blood and melanin content. The results could provide practical insight into better point-of-care treatment with color Cherenkov imaging, as a tool for quantitative dose delivery, independent of the tissue being irradiated.

## 2 Materials and Methods

### 2.1 Tissue Phantoms

**Fig. 1:**
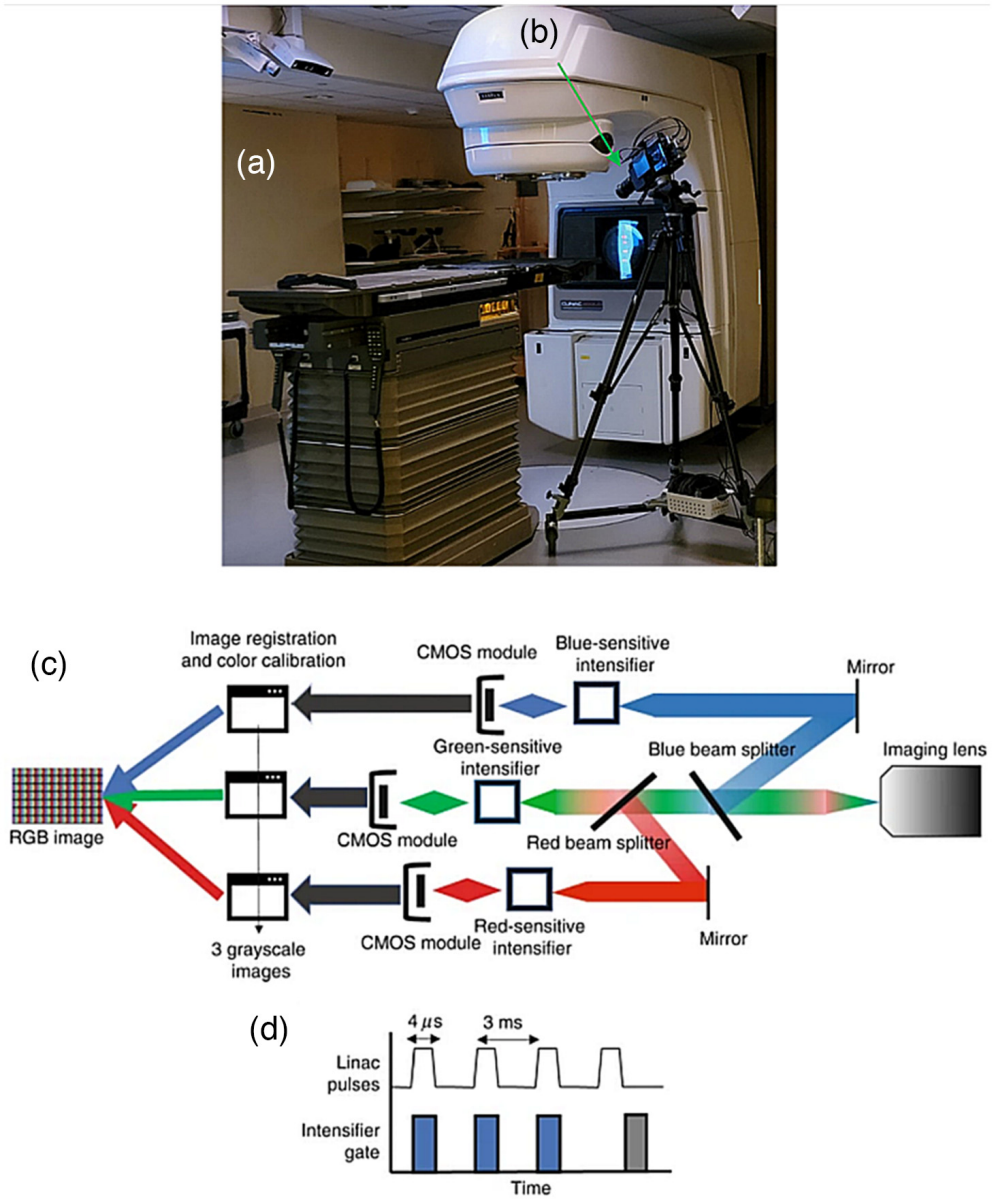
(a) Acquisition setup, using C-Dose software for on-site image processing, LINAC for beam delivery and (b) three-channel time-gated intensified cameras for RGB Cherenkov capture (arrow). Reproduced with permission from Alexander et al. (LSA 2021). (c) Detailed schematic of custom camera.^10^ (d) Representation of necessary time-gating and synchronization to LINAC pulses.^10^

A previously described color Cherenkov camera that captures RGB wavelength channels separately was used in this study.^10^ The acquisition was time-gated to the LINAC, capturing Cherenkov emission only during radiation pulses, thereby removing background ambient light. This setup is shown in Fig. 1(a). Image acquisition and processing were based on prior work,^10^ using the C-Dose research software, which samples image from the camera sensors at fixed numbers of radiation pulses, with the image intensifiers pulsing on with every x-ray pulse from the LINAC and the total image data accumulating on each chip to provide a higher intensity prior to readout to the computer. Background image data was sampled as well and saved onto the FPGA of each camera, such that background subtraction could be achieved on each channel of the camera. Mean intensities for each individual channel were obtained from consistent regions of interest (ROIs) on frame-averaged image stacks.

To simulate tissue, synthetic epidermal layers 100  μm (+/−5  μm) thick were fabricated, with varying biological concentrations of synthetic melanin (M8631, Sigma-Aldrich, St. Louis, Missouri) at 0.0018, 0.0038, 0.0114, 0.019, 0.027, 0.045, and 0.072  mg/ml. Thicknesses were calculated based upon the volume of material produced and the area. The epidermal layers were placed on top of 2-cm thick bulk tissue phantoms made of silicone with flesh-colored pigment, using a process outlined in a previous publication,^12^ composed of silicone embedded with flesh-colored pigments that match human soft tissue optical properties (Smooth-On, Macungie, Pennsylvania).

Melanin concentrations were selected to match the expected range of human skin color based on several factors.^13^^,^^14^ Primarily, we employed the Fitzpatrick scale,^15^ which assessed skin color based on reaction to ultraviolet radiation. Pigmented phantoms representing the following Fitzpatrick scale types were fabricated: Fitzpatrick type 1 (score 0 through 6), type II (score 7 through 13), type III (score 14 through 20), type IV (score 21 through 27), type V (score 29 through 34), and type VI (score 35+). Additionally, we used visual inspection to confirm that the phantoms were within the apparent visual range of human skin color as expected, as we found that the limitation of just the six skin values did not fully represent the range of pigmentation levels existing in human tissue. In particular, at the higher melanin content levels, more delimitation is needed to cover the range of human values. Base concentrations for the pigmented skin phantoms were previously reported.^16^

Average optical properties (absorption coefficient, $\mu_{a}$, and reduced scattering coefficient, ${\mu_{s}}^{\prime }$) of the phantoms were determined to confirm tissue-like optical behavior for a range of human skin colors. These measurements were done with spatial frequency domain imaging (SFDI), explained further below in Sec. 2.6. Intralipid was used as a scatterer at a fixed level of 1%. Bovine whole blood solutions (Lampire Biological Laboratories, Pipersville, Pennsylvania) with varying biological concentrations (0.5%, 1%, 1.5%, 2%, 2.5%, 3%, and 3.5%) were prepared in optically blacked out petri dishes. These values have been used in many previous studies to match the near infrared tissue optical property range for blood absorption in soft human tissues.^7^ Whole blood is fully oxygenated in ambient aqueous solution so that the hemoglobin (Hb) can be assumed to be 100% oxygenated hemoglobin for the spectral signature.

During LINAC irradiation of the phantoms, images were captured for each color channel, and post-processing extracted average RGB Cherenkov emission intensities from the recorded images, as functions of melanin and blood concentrations, as is described below.

### 2.2 Color Cherenkov Camera

The RGB color Cherenkov camera, as seen in Fig. 1(b), was composed of three independent intensified complementary metal oxide semiconductor (iCMOS) cameras (C-Dose, DoseOptics LLC, Lebanon NH) housed in a three-tube color video camera assembly (JVC, Yokohama, Japan). The red channel was outfitted with a red-sensitive intensifier and red filter, the blue and green channels were blue-green sensitive intensifiers, and appropriate color filters for each. Each camera was remotely triggered by leakage x-rays,^17^ allowing for synchronization with and gating to the linear accelerator pulses [Fig. 1(d)]. The camera software supported 16 bit read capability for quantitative acquisition. The beam splitter assembly of the video camera, consisting of RGB dichroic beam splitters and bandpass filters, allowed for incoming Cherenkov light to be redirected according to wavelength to the appropriate camera channel resulting in three raw image stacks for each acquisition, shown in Fig. 1(c). These RGB image stacks were multiple images acquired per imaging time, such that each RGB channel resulted in a temporal image stack for each channel. The camera was equipped with a 10 to 100 mm, f/1.6 zoom lens (JVC, Yokohama, Japan).^10^

### 2.3 Imaged Color Cherenkov Emission

**Fig. 2:**
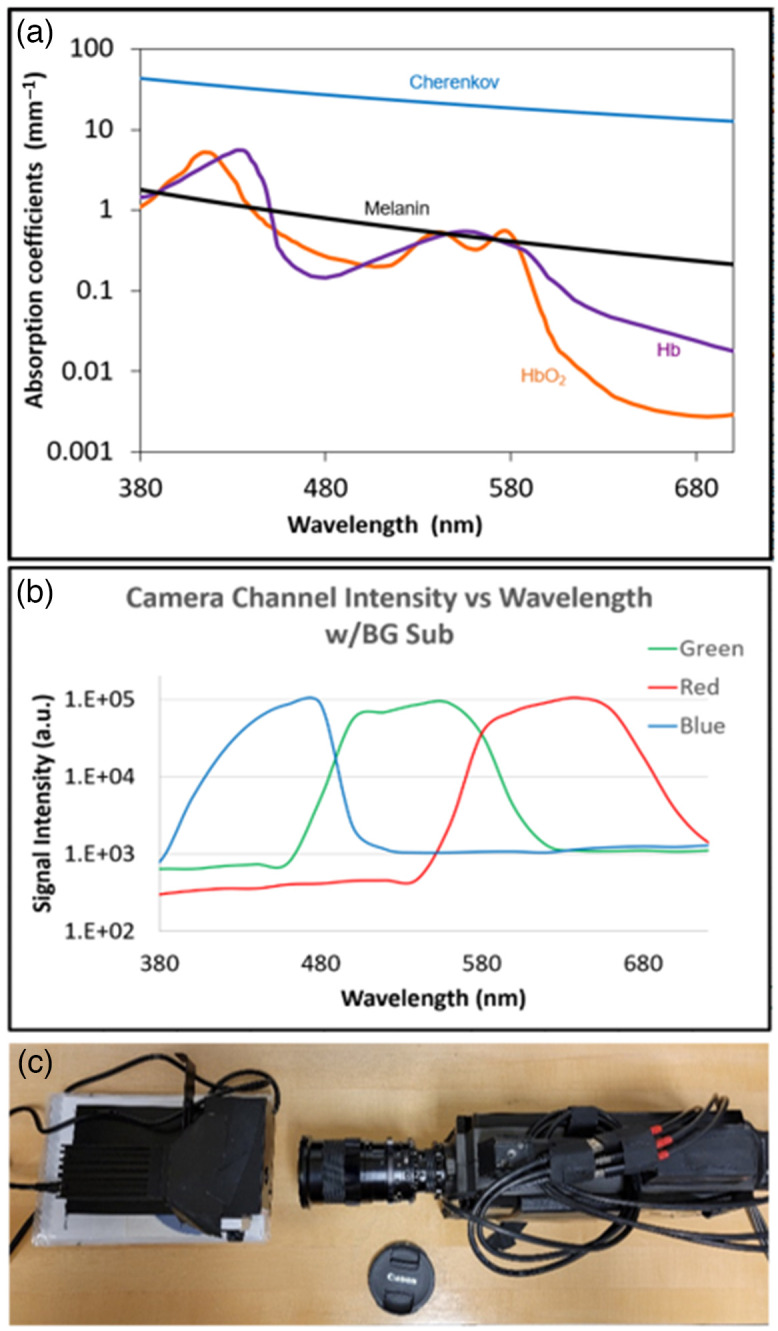
(a) Absorption spectra of melanin, Hb, and ${\mathrm{HbO}}_{2}$ are shown^21^ on the same graph with the emission spectra of Cherenkov, with each arbitrarily scaled. In panel (b) the RGB background subtracted (BG sub) sensitivities of the filtered color Cherenkov camera are shown. (c) A TLS was used to calibrate the camera with narrowband monochromatic light, for the data in (b).

The absorption spectra of melanin,^18^^–^^20^ Hb, and ${\mathrm{HbO}}_{2}$^10^ are shown along with the emission spectrum of Cherenkov light in Fig. 2(a). The sensitivity spectra of RGB channel filters of the RGB color Cherenkov camera are shown in Fig. 2(b). Individual camera detection spectra for the three channels (RGB) were characterized to verify the efficacy and reliability of the filters in tracking and capturing individual channel intensities from experiment tissue samples within expected bands for RGB wavelengths. A tunable light source (TLS) (Optometrics Manual TLS,^22^ Optometrics Manufacturing, Ayer, Massachusetts) was used to characterize the optical system response. Wavelengths from 380 to 720 nm with a step of 20 nm were used for characterization and the response is shown in Fig. 2(b). This was done using a manual TLS and the tri-color camera, shown in Fig. 2(c). The TLS maximized throughput in the visible region of the spectrum using a 20W tungsten halogen lamp, with a spectral energy between 360 and 2000 nm.^23^ The light exiting through a small slit was imaged in close range with the tri-color camera using C-Dose software (DoseOptics LLC, Lebanon, New Hampshire), with images processed in MATLAB (v2022a, Natick, Massachusetts) and signal intensity graphed in Fig. 2(b).

### 2.4 Blood Phantoms

**Fig. 3:**
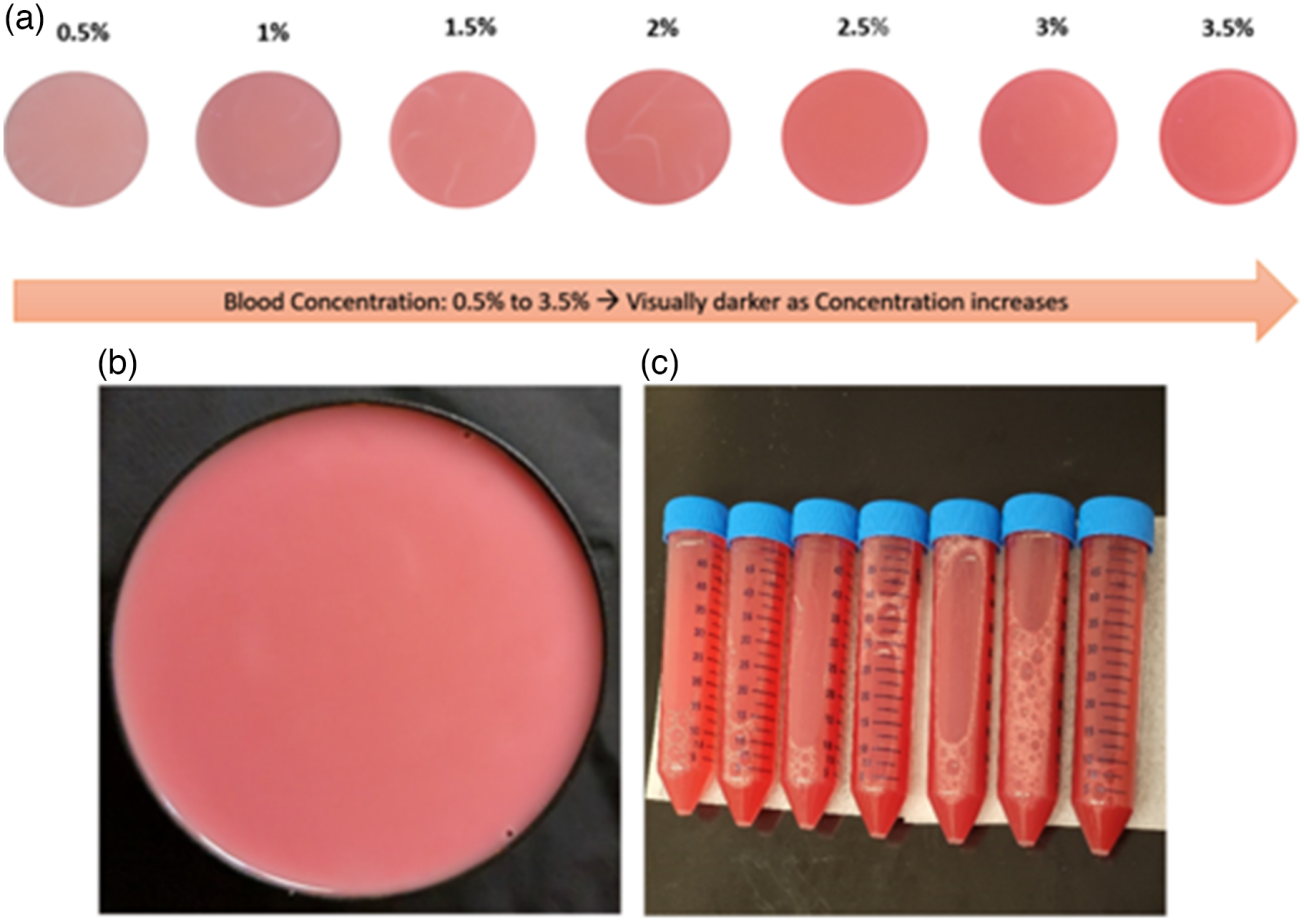
(a) True color images of solutions created in their respective dishes with increasing blood concentration from left to right. (b) Example of single solution dish in blacked-out Petri container. (c) Prepared bovine whole blood and phosphate buffered saline, which were later combined with the Intralipid.

Whole blood solutions at total volume 100 ml were created (Fig. 3) using stock solutions of bovine blood (Lampire Biological Laboratories, Pipersville, Pipersville), phosphate buffered saline solution (Cytiva, Marlborough, Massachusetts), and Intralipid (Sigma-Aldrich, Burlington, Massachusetts). The combined solutions were mixed with concentrations of 1% Intralipid (5 ml at 20% stock emulsion), using variations of blood at 0.5%, 1%, 1.5%, 2%, 2.5%, 3%, and 3.5%, with the remaining volume being phosphate buffered solution. The solutions were poured into 100-mm circular style cell culture dishes, with 20-mm depth [Fig. 3(b)] (Corning, Corning, New York), which had been coated with matte black paint to reduce optical edge effects in the images.

### 2.5 Pigmented Melanin Layers for Phantoms

Synthetic epidermal layers of 0.1-mm thickness were fabricated based on a previously described method.^16^ This was done by dissolving 1 g of gelatin powder of Type A porcine powder, 300 g Bloom (Sigma-Aldrich, St. Louis, Missouri) and 0.5-g glycerol (≥98% purity, Sigma-Aldric, St. Louis, Missouri) in 10 ml of distilled water. Then 0.01% to 0.1% glutaraldehyde (≥99.5% purity, Sigma-Aldrich, St. Louis, Missouri) was added, along with varying concentrations of synthetic melanin (0.0018, 0.0038, 0.0076, 0.019, 0.027, 0.045, and 0.072  mg/ml), which came in the form of small crystals and was manually crushed to a fine powder (M0418-1G Lot# BCCB7179, Sigma-Aldrich, St. Louis, Missouri). The resulting solution was heated evenly with a 1200-W microwave for 5 s to 45°C temperature (Etekcity Lasergrip 1080 IR Thermometer, Anaheim, California) allowing for an even mixture. The solution was poured onto plastic molds (large Fischer weight boats) and then placed into a vacuum chamber to remove air bubbles and distortions. The volume of each melanin mixture was measured such that when it was poured onto the phantom that the layer thickness could be estimated to be 0.1 mm, and was based upon previous trials where after drying the thickness was measured with a micrometer. These layers were then dried for 48 h at 21°C in a fume hood to acquire thin, permanent pliable layers.

### 2.6 Verification of Color Values with Spatial Frequency Domain Imaging

The impact of melanin concentration on the optical properties (absorption coefficient, $\mu_{a}$, and reduced scattering coefficient, ${\mu_{s}}^{\prime }$) of the epidermal layers was quantified using a validated, reflectance geometry SFDI and software (Reflect RS, Modulim, Irvine, California). SFDI^24^ separates the effects of scattering and absorption and can be used to estimate the concentrations of chromophores in the tissue. The technique works by illuminating different patterns light on the tissue, imaging the reemitted light, and demodulating the imaged reflectance.^25^^,^^26^ Optical properties were quantified at each of eight wavelengths (471, 526, 591, 621, 659, 691, 731, and 851 nm) using five spatial projection frequencies (0.00, 0.05, 0.10, 0.15, and $0.20\;{\mathrm{mm}}^{-1}$), and all experimental measurements were calibrated to the supplied tissue phantom from Modulim. The fit to the optical properties of the phantoms used all five spatial frequencies in the inversion, with fit to a lookup table of values within the supplied software. The epidermal layers were placed on top of 2-cm thick bulk tissue phantom made of silicone with flesh-colored pigment during this procedure.

## 3 Results

### 3.1 Average Optical Property Characterization by SFDI

Results from the phantom validation measurements by SFDI are summarized in Fig. 5. Property averages were computed based on the distribution of pixels within each wide-field, circular 5 cm ROI for each phantom. Visual inspection of these optical phantoms, combined with the SFDI property measurements, were used to confirm that the melanin layers exhibited optical properties that corresponded to the expected array of human skin colors.^26^^,^^27^

### 3.2 Cherenkov Images

**Fig. 4:**
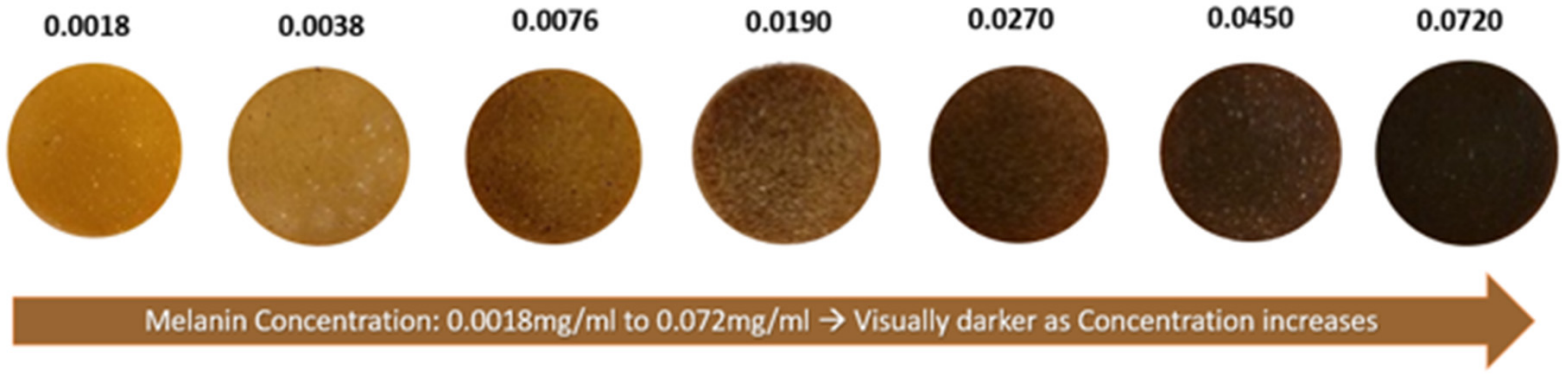
True color view of epidermal layers created in their respective dishes with increasing melanin concentration from left to right.

**Fig. 5:**
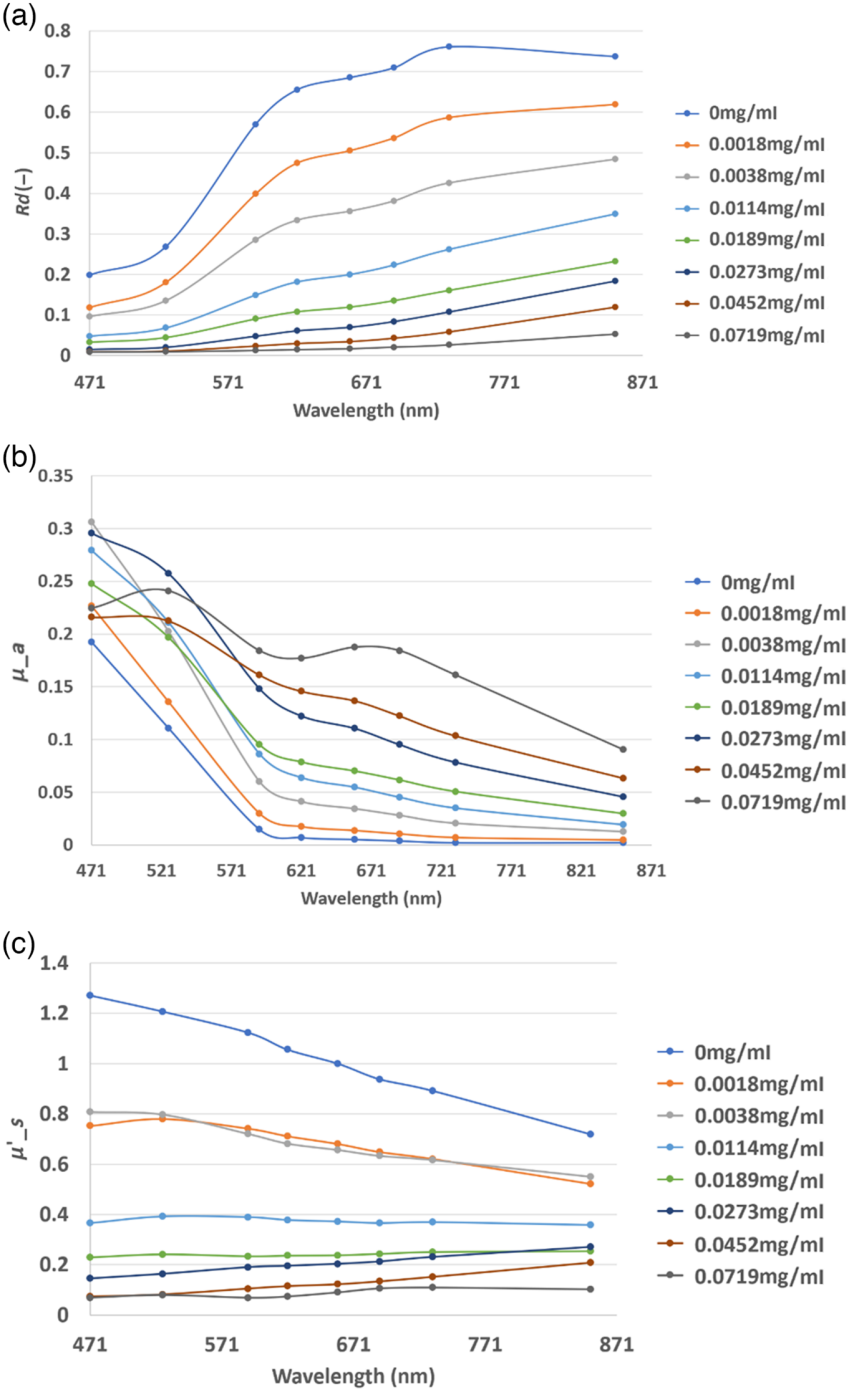
(a) Calibrated reflectance average values; (b) reduced scattering coefficient average values; and (c) absorption coefficient averages, all listed by concentration values in units of mg/ml in the legend.

**Fig. 6:**
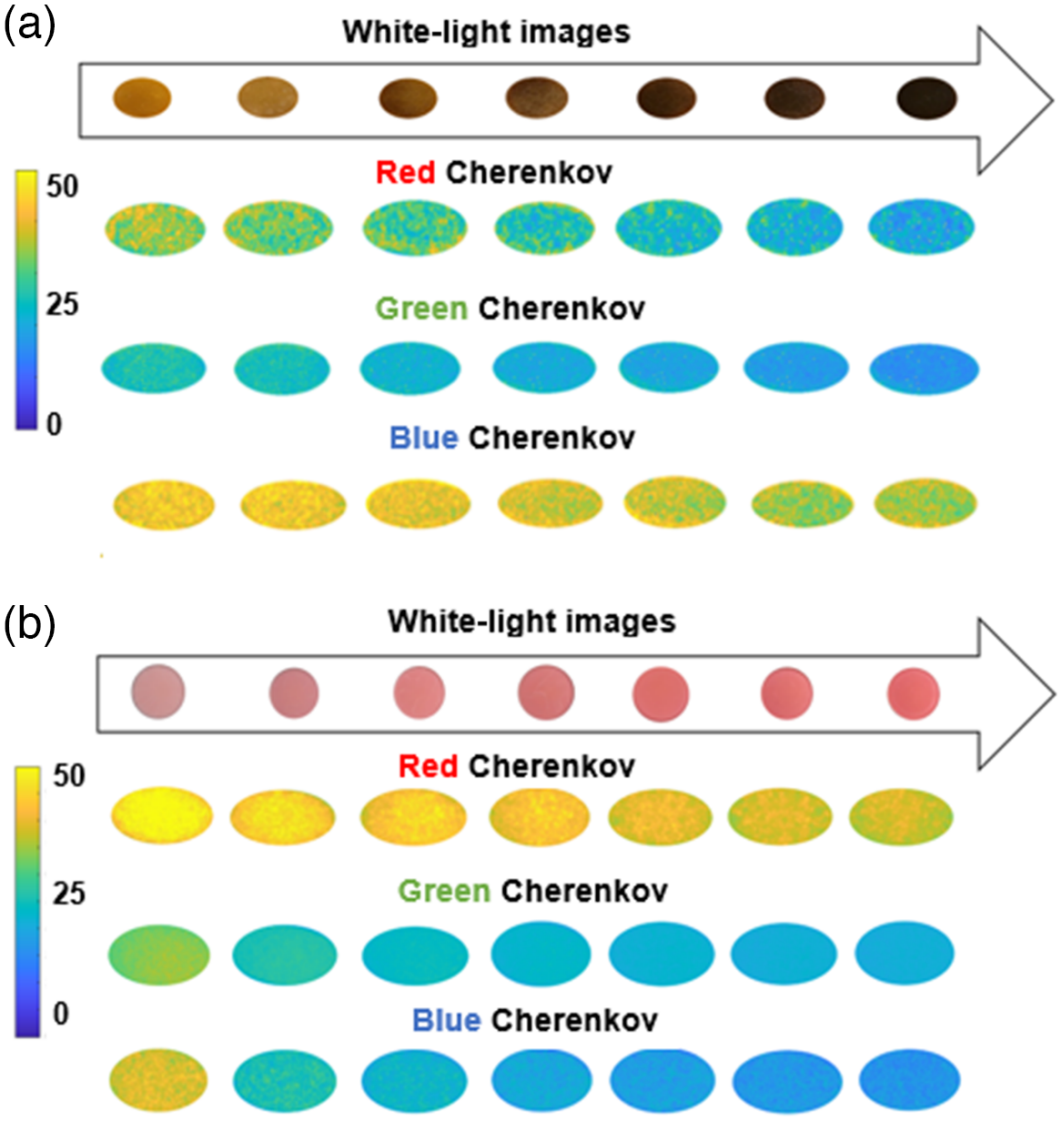
(a) White light images of melanin tissue phantoms with varying absorption. Images of the Cherenkov emission images in each of RGB wavelength bands are shown. Concentrations of melanin were 0.0018, 0.0038, 0.0076, 0.0114, 0.019, 0.027, 0.045, and 0.072  mg/ml, respectively, left to right, with 600  MU/min, 6 MV and 300 MU (b) White-light images of blood phantoms with varying absorption. Images of the Cherenkov emission images in each of RGB wavelength bands are shown. Concentrations of blood were 0.5%, 1%, 1.5%, 2%, 2.5%, 3%, and 3.5%, respectively, left to right, with 600  MU/min, 6 MV, and 300 MU.

RGB resolved Cherenkov images for varying melanin and blood concentration variations are shown in Fig. 6 and are compared to white light images. The attenuation in Cherenkov emission from tissue phantoms due to melanin absorption is shown in Fig. 6(a). Emission was observed to decrease as melanin concentration increased, without one particular color vastly differentiating itself in intensity from another. This is in line with melanin spectral properties showing no anomalous preference to a particular wavelength and decreasing in absorption units with increasing wavelength as noted in literature^28^^–^^30^ as well as being confirmed via SFDI optical property validation in Figs. 4 and 5. For purposes of visualization, the blue channel needed individualized windowing and leveling, and thus, a separate color bar is shown. The zero concentration of melanin was a gelatin layer with no melanin present in it.

The attenuation in Cherenkov emission from the blood samples is shown in Fig. 6(b). The observed attenuation increased with increasing blood concentration with the red channel showing the greatest in mean signal intensity change compared to the green and blue channels. This is in line with blood spectral properties showing preference to a particular wavelength—Hb bound to oxygen absorbs blue-green light, reflecting red-orange light^31^—appearing red and varying non uniformly in absorption units with increasing wavelength as noted in literature.^28^^–^^30^ For purposes of visualization, as before, the blue channel needed individualized windowing and leveling and thus a separate color bar is shown.

### 3.3 Differential Signal Response

**Fig. 7:**
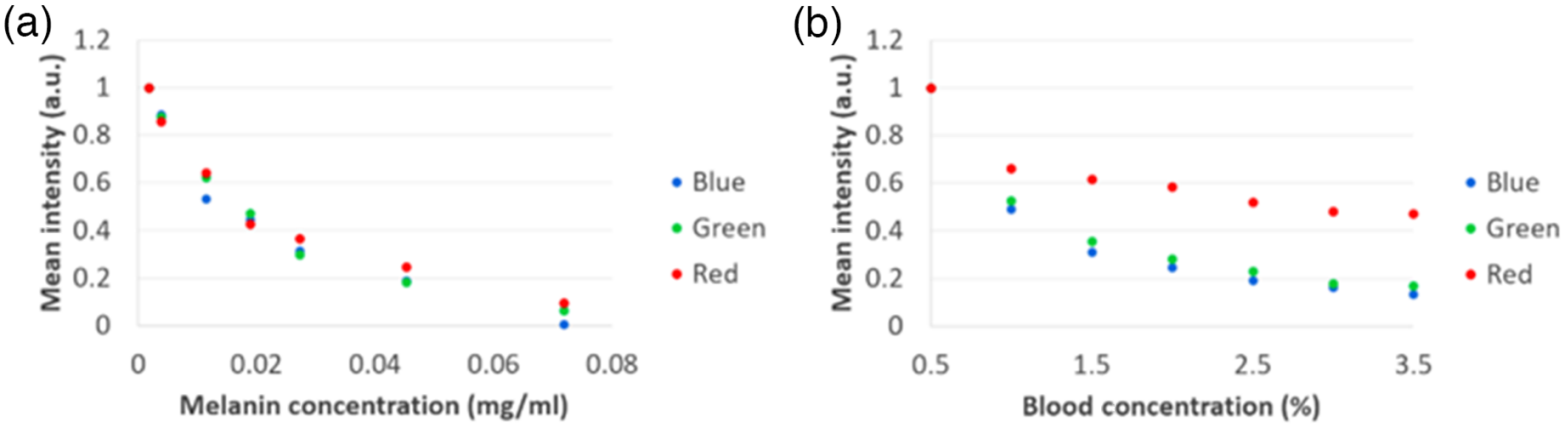
Normalized Cherenkov RGB signal intensities versus (a) melanin concentration and (b) blood concentration.

Cherenkov images from each of the individual channels—gathered using C-Dose software and processed in MATLAB—resulted in the output of signal intensities across various concentrations in melanin and whole bovine blood, as shown in Fig. 7. Data gathered show an attenuation response for both melanin and blood. For melanin, as per visual aid of Figs. 6(a) and 6(b), the quantitative response graphed in Fig. 6(a) shows all channels following lower emission intensity with increasing pigment concentration. Comparing this response with the reference chart in Fig. 2(a), the trend seen in our results follows what is expected for Cherenkov emission and melanin concentration. Conversely, the whole bovine blood quantification [Fig. 7(b)], with visual aid from Figs. 6(a) and 6(b), shows a differential response from the three channels. The red channel Cherenkov signal intensity attenuates to a much lesser extent, while the green and blue channels follow a tight diminution together to levels lower than red as a whole. Repeated measures of the phantoms confirmed these observed trends, with the error bars being smaller than the data points themselves. The increased absorption of blue and green by Hb^31^ results in increased signal in the red channel. Furthermore, Fig. 2(a) corroborates this observation; oxygenated Hb exhibits two peaks where blue and green light wavelengths are represented.

This phenomenon of different amounts of exiting light in the RGB bands in color Cherenkov imaging provides quantitative and qualitative confirmation of detection of signals that varied differently with changes in blood or melanin content.

**Fig. 8:**
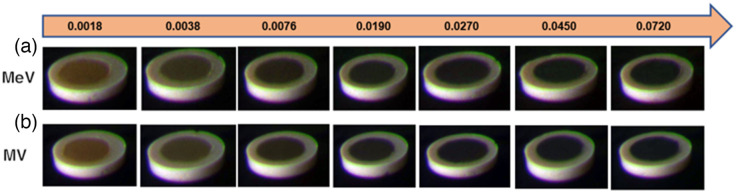
Visual display of seven phantoms chosen to show slight variation in melanin content at the lowest values. The standard color photograph of the tissue phantoms is shown in Fig. 4 with ambient room lighting, and the Cherenkov color image is shown here in (a) and (b) taken in a darkened room with (a) MeV beams and (b) MV beams. The values depicted above the images are in units of mg/ml to represent biological melanin concentration in each phantom.

Further color Cherenkov imaging of skin pigmentation phantoms is seen in Fig. 8. About 3 Gy of dose was given via 6-MV photon and 6-MeV electron beams to the seven phantoms to show the limits of visual analysis of Cherenkov color imaging. With both beam energies, pigmentation past 0.0076  mg/ml is seen to be extremely difficult to distinguish from the background—effectively no observable Cherenkov emission. This observation also further illustrates the need for correcting Cherenkov signal attenuation due to biological tissue factors so that the emission can be seen as visually independent from patient-specific attenuating influences. It is noteworthy that the zero concentration values were removed because of reflection effects in the samples that placed their values outside of the trend with expected human tissues. Given that these concentrations are unrealistic of human tissues, their removal was deemed appropriate and provided a clearer interpretation of the trends seen here.

## 4 Discussions

This study examined the expansion from prior work on Cherenkov imaging^7^^,^^10^^,^^12^^,^^13^ but with the use of a three-channel RGB Cherenkov emission camera, to illustrate how imaging in color may provide a visual separation of tissue attenuation effects. The rationale for this study was to better understand how tissue optical properties affect the emission colors of Cherenkov light, to determine if there might be ways to use the spectrum for calibration or correction for tissue attenuation effects. The hypothesis driving this work was that differential RGB color Cherenkov emission levels would result from variations in the most dominant biological tissue absorption features, such as blood concentration within tissue and melanin concentration in the skin. Experimental measurements shown in Figs. 6–8 visually demonstrate that this is true. Furthermore, these changes in blood or melanin concentrations result in distinct visual changes in the RGB outputs values of the Cherenkov color emission. These findings support the idea that color or spectral imaging of Cherenkov might provide an experimental methodology for separation of biological attenuation of the intensity from the physical generation of Cherenkov with dose deposition. The goal would ideally be to use the Cherenkov intensity as an indicator of the dose delivered in the tissue, independent of the blood volume within it or the skin color, using color correction.

While spectroscopic imaging of Cherenkov would likely provide a better quantitative measure of the color changes, the utility and convention of imaging in three RGB channels is ubiquitous today. The images provide a visual cue to the users of Cherenkov imaging about the biological origins of what is being seen. It is possible that corrected Cherenkov images could be displayed, side by side with information about the melanin or blood levels. Oxygenation studies have been examined in a previous paper,^10^ but here the focus was maintained on oxygenated blood. The light penetrance and imaged depth will vary with color, as a side effect of the attenuation values, meaning that blue and green light will likely have less than a mm penetration, while red light may have up to 1- to 5-mm penetrance. The penetrance is determined by the absorption and scattering coefficients at each wavelength band for this light, and the escape is exponentially attenuated with depth. So the average emission depths quoted here are simply average value for escaping photons that are distributed from an exponentially weighted depth of escape from the tissue. Major variations in depth will only be a large issue in tissues that have a highly layered variation with depth and might cause issues in areas of scar, tattoo, or burn, which are strongly different from the surrounding normal tissues.

Perhaps one of the most central questions from this work is: how much attenuation occurs from skin color when acquiring Cherenkov images? The attenuation depends upon the concentration of melanin in the skin, and examining the data in Fig. 7(a) and the images in Figs. 8(a) and 8(b) provide the answer to this. The skin colors imaged in the phantom correspond to the range of human skin colors expected, with the darkest having an extremely high melanin level, and in the data shows a 90% to 100% reduction in Cherenkov emission in all three color channels, with blue and green being the least emitted. It may not be possible to perform Cherenkov color imaging in people with the darkest skin tone because of the extreme emission attenuation, visually shown in Fig. 8. It should be noted that despite this, imaging in the next lower melanin level was possible with attenuation just at the 75% to 80% level. An increase in camera or image gain might be employed to overcome this.

A major advantage to the setup in this work is the capacity to quickly and efficiently gather images showing contrast between changing biological factor concentration (blood and melanin here) with a portable setup. Future studies might examine and confirm if the separate effects of melanin and blood are indeed independent and separable when combined in the same phantoms. Based upon the fact that there was a monotonic response seen in intensity variation with melanin concentration (see Fig. 7) and that there was independent information seen in the three color channels between melanin and blood volume, it seems like it would be possible to provide a linear correction factor for the attenuation due to melanin and blood volume when imaging in vivo, by developing a 2×3 matrix where the three normalized intensities of RBG are fitted to values of melanin and blood volume, and an inversion solved for correction of the corrected Cherenkov intensity.

Scattering effects were not focused on here, largely because they are not as dominant a change between individuals and across individuals as much as blood or melanin does, as shown in previous studies.^27^ Hb content can vary by a factor of two between tissue types, and melanin can vary by a factor of 10 across individuals, and so these variations were thought to be most dominant. While there are large estimated scattering changes in the melanin phantoms here [Fig. 5(b)], these are likely erroneous due to the SFDI measurement being very surface weighted.^32^ The light fluence escape from tissue is dominated by the bulk tissue absorption and scattering coefficients, and the thin layer of melanin will appear as a thin attenuation filter for the emission light coming out, but not necessarily as a variation in the bulk tissue scattering coefficient. However future studies might examine the subtly of how much the normal variations of scattering might affect the Cherenkov color intensity measurement, and how layers of varying scatter might affect the signal independent of layers of absorption.^33^^,^^34^

Future work would include automation of image processing as well as further investigation of correction factors for variation in blood and melanin concentration changes in patients, as well as correcting for lighting conditions and camera setup to ensure highest possible signal-to-noise ratio in fully color-resolved Cherenkov image output. The work could also be expanded to study the variances in RGB color Cherenkov imaging colors, comparing entrance and exit beams as well as MV and MeV energies. This would be interesting since the apparent color may likely change because the buildup curve is different for these two types of radiation, and hence spectral attenuations would likely be different.

Patient studies could be performed on a large-scale clinical level, with a focus on the impact of how differential Cherenkov signal response contributes to Cherenkov dose readouts and guide better, more accurate patient surface dosimetry. This would require the manufacturing of an equal or better camera setup if it is to be done in multiple locations, and involve a careful step-by-step acquisition procedure with consistency in room and camera setup. Such future work would be done with a greater diversity of patients as opposed to previous studies done with mainly lower melanin pigmentation as well as varying levels of blood concentration or oxygenation in accordance with any present afflictions or ailments resulting in perturbation from a basal state.

Further work could examine the context of other biological factors such as lipids and localized blood in vasculature. A whole skin model could potentially be used to further this goal, with tunable sections to monitor the effects of changing variables, allowing for a major expansion to this work on blood and melanin alone. It is now becoming more widely known that the skin color effects can alter emitted light signals,^11^ and so, it is possible that quantitative spectral measurements might be better utilized to compensate for these types of attenuation effects, and provide better information about the underlying light signals emitted from deeper in the tissue.

## 5 Conclusions

This study quantitatively and visually showed how the imaging of biological tissue using RGB color Cherenkov imaging could provide independent information on intensity variations from pigmented skin or Hb level variations in the material, with this information being independent of the total dose. The differential signal response seen in blood versus melanin shows that it would be possible to differentiate the attenuation effects of the two spectrally. There is a possibility that future studies could use this information to correct Cherenkov intensity for the attenuation of one of these biological factors. However, an important observation is also that in the very darkest color phantoms, it seems as though there may be insufficient blue Cherenkov light emitted to gain a reliable signal without large improvements to the light capture approach. Still red wavelengths were sufficient in all skin color phantoms, albeit with a near 90% reduction in the darkest skin tones. Further focus on spectral distortion corrections for Cherenkov intensity changes might be used in quantitative patient dosimetric imaging.

## Code, Data, and Materials Availability

The data produced in this report are available to interested readers upon reasonable request to the corresponding author. No original software was produced in this effort.
